# Needs Expressed in Peer-to-Peer Web-Based Interactions Among People With Depression and Anxiety Disorders Hospitalized in a Mental Health Facility: Mixed Methods Study

**DOI:** 10.2196/51506

**Published:** 2024-07-12

**Authors:** Dawid Storman, Paweł Jemioło, Zuzanna Sawiec, Mateusz Jan Swierz, Ewa Antonowicz, Malgorzata M Bala, Anna Prokop-Dorner

**Affiliations:** 1 Chair of Epidemiology and Preventive Medicine Department of Hygiene and Dietetics Jagiellonian University Medical College Kraków Poland; 2 AGH University of Krakow Kraków Poland; 3 Students’ Scientific Research Group of Systematic Reviews Jagiellonian University Medical College Kraków Poland; 4 Chair of Epidemiology and Preventive Medicine Department of Medical Sociology Jagiellonian University Medical College Kraków Poland

**Keywords:** anxiety disorders, depression, peer-to-peer web-based interactions, needs, psychiatric hospitalization

## Abstract

**Background:**

Hospitalization in psychiatric wards is a necessary step for many individuals experiencing severe mental health issues. However, being hospitalized can also be a stressful and unsettling experience. It is crucial to understand and address the various needs of hospitalized individuals with psychiatric disorders to promote their overall well-being and support their recovery.

**Objective:**

Our objectives were to identify and describe individual needs related to mental hospitals through peer-to-peer interactions on Polish web-based forums among individuals with depression and anxiety disorders and to assess whether these needs were addressed by peers.

**Methods:**

We conducted a search of web-based forums focused on depression and anxiety and selected samples of 160 and 176 posts, respectively, until we reached saturation. A mixed methods analysis that included an in-depth content analysis, the Pearson *χ*^2^ test, and φ coefficient was used to evaluate the posts.

**Results:**

The most frequently identified needs were the same for depression and anxiety forums and involved informational (105/160, 65.6% and 169/393, 43%, respectively), social life (17/160, 10.6% and 90/393, 22.9%, respectively), and emotional (9/160, 5.6% and 66/393, 16.8%, respectively) needs. The results show that there is no difference in the expression of needs between the analyzed forums. The needs were directly (42/47, 89% vs 98/110, 89.1% of times for depression and anxiety, respectively) and not fully (27/47, 57% vs 86/110, 78.2% of times for depression and anxiety, respectively) addressed by forum users. In quantitative analysis, we found that depression-related forums had more posts about the need for informational support and rectification, the expression of anger, and seeking professional support. By contrast, anxiety-related forums had more posts about the need for emotional support; social life; and information concerning medications, hope, and motivation. The most common co-occurrence of expressed needs was between sharing own experience and the need for professional support, with a strong positive association. The qualitative analysis showed that users join web-based communities to discuss their fears and questions about psychiatric hospitals. The posts revealed 4 mental and emotional representations of psychiatric hospitals: the hospital as an unknown place, the ambivalence of presumptions and needs, the negative representation of psychiatric hospitals, and the people associated with psychiatric hospitals. The tone of the posts was mostly negative, with discussions revolving around negative stereotypes; traumatic experiences; and beliefs that increased anxiety, shock, and fright and deterred users from hospitalization.

**Conclusions:**

Our study demonstrates that web-based forums can provide a platform for individuals with depression and anxiety disorders to express a wide range of needs. Most needs were addressed by peers but not sufficiently. Mental health professionals can benefit from these findings by gaining insights into the unique needs and concerns of their patients, thus allowing for more effective treatment and support.

## Introduction

### Background

Over recent decades, the prevalence of depression has risen globally, affecting approximately 280 million people in 2019 [1]. Major depressive disorder is often characterized by recurrent episodes and imposes substantial challenges, including social difficulties, increased health care use, diminished work productivity, and heightened mortality risk [2,3]. The COVID-19 pandemic exacerbated mental health issues, leading to a 25% surge in depression and anxiety rates worldwide, including among various populations in Poland [4-9]. During mental health crises, individuals may seek help independently or from others, encountering barriers in accessing professional psychiatric care, especially pronounced in countries such as Poland with a shortage of psychiatrists and other specialists [10-15]. Web-based forums provide a less stigmatizing platform for seeking mental health information and support, enabling users to interact with peers and access resources anonymously. Other key advantages of web-based mental health forums include anonymity, easy access, continuous availability, the option for passive content consumption, peer-to-peer type of interaction, informality compared with a physician-patient relationship, and free accessibility [16].

However, misinformation on social media presents risks, with 40% of shared health-related links being categorized as fake news [17]. Using web-based platforms for mental health support has become increasingly common, facilitating knowledge exchange, emotional support, and community engagement [18]. These platforms also regard the topic of hospitalization in mental health facilities.

There are not many strict indications regarding who should be hospitalized in a psychiatric hospital, and these indications may vary by country [19-21]. However, it is generally agreed that hospitalization is needed when a patient experiences an exacerbation of the disease and presents a risk to themselves or others [19]. The World Health Organization Division of Mental Health and Prevention of Substance Abuse published 10 basic principles of mental health care law [22], such as the promotion of mental health, access to basic mental health care, guidelines on assessing necessary treatment options, and patient autonomy, along with suggestions on selected actions to promote the implementation of these principles. However, not all countries follow or implement them.

Other indications for hospitalization include a rapid or severe deterioration of the patient’s mental state, a skewed perception of reality, an inability to handle the patient’s condition in the ambulatory setting, or the need for the patient to leave the home environment [19,23]. However, hospitalization can cause shock and trauma in patients, especially if it is forced. Therefore, such decisions should always be thoroughly considered [24]. In the context of psychiatric hospital admissions, there are generally 2 primary ways through which individuals can be admitted: voluntarily or involuntarily. Voluntary admission occurs when an individual willingly seeks treatment and agrees to be admitted to a psychiatric hospital. They may recognize the need for assistance with their mental health and voluntarily choose to seek help. By contrast, involuntary admission takes place when an individual is admitted to a psychiatric hospital against their will. Involuntary admission typically happens when someone’s mental health condition poses a significant risk to themselves or others and they are unable or unwilling to seek help voluntarily. Involuntary admission usually involves legal procedures and may require the involvement of mental health professionals, law enforcement, or the judicial system. Across the European Union, the frequency of involuntary admissions to psychiatric hospitals varies considerably, ranging from approximately 3% to 30% of all inpatient episodes [25].

The rules and regulations for involuntary admission and treatment of patients with mental disorders differ markedly across countries [26]. Essential legal criterion for detention, in addition to mental disorder in most European countries (eg, Poland, Austria, Belgium, France, Germany, and the Netherlands), is a threat or an actual danger to one’s own life or others’ safety, the need for treatment (eg, Italy, Spain, and the United Kingdom), or both (eg, Denmark, Finland, Greece, and Ireland) [27]. In case of the maximum length of initial hospitalization, Italy and Poland are characterized by the shortest period (7 days and 10 days, respectively), while Belgium and Germany allow for the longest hospitalization (up to 2 years). In Poland, involuntary admission to a mental health facility is regulated by the Mental Health Act of 1994 (in Polish, Ustawa o ochronie zdrowia psychicznego). It is allowed in the following situations: (1) when the patient experiences an exacerbation of the mental state and poses a risk to themselves (article 23); (2) when the patient develops symptoms of a new disease and is admitted for diagnostic workup, just for observation, without pharmacotherapy, for a maximum stay of 10 days (article 24); and (3) when the court orders compulsory hospitalization (article 29) [28]. Admission to a psychiatric hospital is regulated by the Convention on the Rights of Persons with Disabilities drafted in 2006 [29]. Up to February 21, 2023, a total of 186 countries, including Poland, gave their explicit consent to be bound by the treaty [30]. The convention has a strong position on hospitalization without consent, and Poland is repeatedly reprimanded for failing to comply with its provisions [31].

The length of stay in a psychiatric hospital can vary depending on the severity of the mental condition and response to treatment, and again, this differs significantly between countries [32,33]. The mean time of hospital stay for any mental disorder ranges from 6.8 (SD 3) to 55.1 (SD 62.4) days [34-37]. On average, hospitalization is the longest in patients with schizophrenia, ranging from 21 to 290 days [38,39]. For patients with dual diagnosis, the mean reported hospitalization duration was 12 days [38]. The mean length of hospital stay for manic, depressive, and mixed episodes was 29.2, 29.9, and 43.3 days, respectively [40]. Finally, according to the literature, factors associated with longer hospitalization include relationship status (being single) and unemployment [41].

After every hospitalization, patients need to return to their regular environment. To facilitate this transition, the time of hospital treatment should be reduced, and dedicated programs should be available in the outpatient setting. The process of change that led to the deinstitutionalization of psychiatric care began in Western Europe and the United States in the 1950s. The revolution in psychopharmacology had a significant impact on these transformations, as it improved the effectiveness of treatment, allowing many patients to function outside the hospital setting. Pioneering attempts at new therapeutic methods began in Anglo-Saxon countries; France promoted psychiatry without psychiatrists, and the United States started preparations for the deinstitutionalization process. New legislation was introduced to foster a noninstitutional approach to psychiatric care (1946-1959 in the United Kingdom and 1955-1963 in the United States). In France, the sectorization system was introduced in the 1960s [42]. In Italy, reforms were started by the representatives of a new European generation that was critically oriented toward the revolution of psychotropic drugs and psychoanalysis and was fascinated by phenomenology, poststructuralism, and Marxism. The most radical current emerging from these attitudes was called *antipsychiatry*. The 1960s were the times of increasing left-wing and antiauthoritarian social moods. Controversies around psychiatry resulted in a tendency to question its traditional prerogatives. A trend that saw psychiatry as an oppressive element of the capitalist system emerged. The developing ideas of antipsychiatry corresponded well with this atmosphere, and around 1968, they became an important counterculture and political current. While British, French, and American antipsychiatrists contested the psychiatric care system, radical Italian psychiatrists, led by Basaglia, attempted to dismantle it from within and effectively created social support for the deinstitutionalization process [43]. In Germany, many hospital wards were closed as a result of a psychiatric reform, and a net of health care professionals providing integrated care was created. This form of treatment offers the possibility of treating more patients by applying different treatment intensities and contents [44].

Depending on their mental condition and the ability to function in day-to-day activities, patients can be admitted to an inpatient facility (eg, a hospital ward, psychiatric hospital, residential house, or, in specific cases, a high-security forensic unit), or treatment can be provided in the outpatient setting, with daily visits to the hospital without an overnight stay. However, hospitalization is the best way to control a patient’s behavior and adjust treatment [45,46]. During psychiatric hospitalization, a person typically receives a range of mental health treatments, such as medication, psychotherapy, and support from a multidisciplinary team of mental health professionals. However, hospital wards are not always perceived as a safe space. Patients face challenges as they adapt to new conditions; cope with restrictions regarding visits and leaving the ward; and deal with other patients or personnel at the facility, where mistreatment, aggressiveness, theft, or substance abuse take place [47,48]. There have been numerous cases of mistreatment by staff members or other patients. Examples of such behaviors include physical and verbal abuse, neglect, and isolation. In some cases, staff members may go too far when using restraints or other forms of physical intervention, causing harm to patients [49-51].

### Objectives

The role of the media in reinforcing mental health stereotypes and stigmas is an important issue. For example, some news outlets tend to sensationalize incidents of violence or other disruptive behaviors, thus portraying patients as dangerous or unstable. This can create a climate of fear and mistrust toward patients with psychiatric disorders, making it even harder for them to receive the care and support they need [52-55]. Alzahrani [56] suggested that there have not been enough studies that identify and examine the effect of hospitalization in a mental health facility on the emotional status and well-being of patients. Unmet needs are particularly problematic in patients with psychiatric disorders because their mental condition might be an obstacle to expressing these needs [57,58]. One such condition is dementia, as studied by Røsvik and Rokstad [59] in a systematic review aimed at identifying the needs of patients, caregivers, and staff in the context of acute hospitalization. There have been studies conducted with the objective of deepening our understanding of patients’ needs. They concern identifying needs by professionals [60], a sense of safety during psychiatric care [61], or a feeling of being cared for by nurses [62]. However, it is not common for investigators to examine the needs of people in the hospital setting via social media. Therefore, the aim of this study was to fill in this gap by identifying and exploring the needs of people regarding hospitalization in mental health facilities via internet forums.

## Methods

### Overview

For this study, we used a mixed methods design (qualitative and quantitative methods). To ensure the scientific rigor and transparency of our research, we adhered to standards for reporting qualitative research and qualitative meta-analysis, which provide systematic guidelines for describing research questions, study design, data collection and analysis, findings, and study implications [63]. Methods used in this study are briefly described in the subsequent sections. Detailed methodology was reported previously (Storman, D, unpublished data, May 2024).

### Data Collection

We conducted a search for Polish-language web-based forums dedicated to individuals with depression and anxiety disorders. We used the following search terms in Polish: “forum” (in English, forum), “depresja” (depression), and “zaburzenia lękowe” (anxiety disorders) and scanned the first 10 pages of Google (Google LLC) search results. Of the 10 identified forums, we selected 2 (20%) forums with the highest number of registered users and posts: one forum related specifically to depression (depresja forum) and the other related to anxiety disorders (nerwica forum). Posts were obtained using a web scraping technique with Python (Python Software Foundation) code on July 18, 2021.

The selected forums were free of charge, open to the public, noncommercial, and nongovernmental. In 2005, the depresja forum [64] was established for individuals experiencing depression, while nerwica forum [65], also established in 2005, was intended for people with anxiety disorders. The statute of the forums indicates that the key objective is to facilitate anonymous interactions and mutual support among individuals experiencing depression or anxiety disorders. The content is monitored by volunteer peers who ensure adherence to forum rules. The users were free to open threads and answer them. The 2 selected forums did not require users to register to create a discussion thread, post comments, or reply to comments from other users. Forum discussions were asynchronous, with posts appearing in sequence, which means that the simultaneous presence of all participants in the thread was not necessary. The moderators of the forums did not provide any therapeutic interventions. While individuals without personal experience of depression or anxiety disorders, such as family or friends, could participate in the forums, only posts indicating that their authors had personal experience with mental disorders were considered for the analysis.

### Sampling

We sampled the posts (160 from depression forums and 176 from anxiety forums) until achieving saturation of the data, that is, the content provided rich, detailed information relevant to the research question, and no new data were obtained in the following posts. We did not impose any limits regarding time, that is, when the post was published or which stage of hospitalization (before, during, or after) its content considered.

### Coding

We used a deductive coding approach to assess the selected material, based on a codebook developed in the previous study (Storman, D, unpublished data, May 2024). Our unit of coding was a single interaction, consisting of a presentation of a need (which could be found in the opening post or within the discussion) and a response (posts within the same forum thread where the authors quoted a specific post, mentioned a specific user, or referred to a specific content). The unit of analysis was a forum thread. We considered only threads with at least 2 posts, as this was the minimum number of posts required to observe interactions. We coded the interactions between forum users that revealed their needs related to psychiatric hospitalization. Every post was coded based on how the need was presented: explicitly (a direct question) or implicitly (a problem disclosed indirectly). We also coded how peers responded to the needs, categorizing responses as fully or not fully addressed (eg, off topic) and as direct or indirect (eg, referring to the asker in the third person). We evaluated the adequacy of responses only when it was indicated by the asker, such as by thanking the responder or by expressing dissatisfaction with the response.

### Reliability

To ensure coding quality, 4 coders underwent coding training with an experienced qualitative researcher involved in the study. Independent coders achieved an agreement rate of >70%. Any disagreements were resolved through discussion.

We began by immersing ourselves in the data to gain a comprehensive understanding of the content of the posts. Each coder kept a logbook to record their observations and insights as they worked through the data. After coding the data by 2 independent coders and comparing the coded fragments across different users, threads, and forums, we aggregated segments of posts that were coded with the same code and compared them within and between cases. This enabled us to identify patterns and variations in the data, identify the main themes, and gain a deeper understanding of the main themes and trends in the posts. Throughout the analysis, we wrote summaries and memos to document our observations, hypotheses, and insights, which helped us better understand the emerging patterns.

Our team included interdisciplinary investigators with different professional backgrounds, such as medicine and health sciences (including psychiatry, epidemiology, and public health), psychology, and sociology, which enriched our discussions and reflexivity throughout the study. The coding team convened on multiple occasions to ensure a shared understanding of the material. Upon the completion of the coding process, the first author continued with subsequent analysis steps. To foster reflexivity, routine group discussions were conducted throughout the study, focusing on scrutinizing premature observations and challenging underlying assumptions.

### Analysis

In this study, we used a mixed methods approach to content analysis. Qualitative content analysis was used to understand experiences presented in the posts, while quantitative analysis was used to provide numerical descriptions of the data and explore relationships between needs and trends. We coded the interactions between forum users that revealed their needs related to hospitalization in psychiatric units using a codebook developed in our previous study [66]. Coding quality was ensured by training all the coders. Any discrepancies were resolved through discussion. The qualitative analysis was conducted by following the approach suggested by Krippendorff [67] and using MAXQDA 2022 (VERBI Software) [68,69], a qualitative data analysis software, for coding, annotating, retrieving, and reviewing textual data.

Using the SPSS (IBM Corp) software [70] to determine whether there is a significant association between the groups (forum members experiencing depression and anxiety disorders), the Pearson *χ*^2^ test was applied, with the significance level set at *P*<.05. The co-occurrence of codes was presented using a circular chart (co-occurrence frequencies) to investigate possible associations between interactions. The graph was prepared using NetworkX (version 2.5.1; Los Alamos National Laboratory) for Python. We used the φ coefficient [71] to examine the associations between the types of different interactions.

To differentiate the authors of the posts, we used 2 criteria: self-reported gender (woman, man, or unknown) and the level of activity on the forum based on the number of posts published. The categories of activity levels were defined as follows: 1 to 500 posts: occasional user; 501 to 1000 posts: regular user; 1001 to 5000 posts: super user; and >5000 posts: power user. For those who were not registered on the forum and remained anonymous (guests), it was impossible to determine the level of activity. Therefore, their level of activity was not reported.

### Ethical Considerations

On the basis of the guidance and ethical recommendations of the Secretary’s Advisory Committee on Human Research Protections, our study did not require ethics approval and informed consent because it involved the collection and analysis of publicly available information from internet forums. All data collected in this study were anonymized and deidentified. The study was strictly archival and cross-sectional in nature, without any intervention or interaction with the forum users, and only used preexisting information [72-74].

## Results

### Quantitative Description of the Material

#### Overview

The posts included in this analysis were published between 2013 and 2021. The mean length of coded posts from the anxiety-related forum was 485 (SD 477; range 24-2255) signs and that from the depression-related forum was 518 (SD 691; range 41-5773) signs. A detailed description of the forums was compiled previously (Storman, D, unpublished data, May 2024). In this study, we focused exclusively on posts discussing psychiatric hospitalization.

The frequencies of codes and their descriptions are presented in Table 1 (main codes) and Table S1 in Multimedia Appendix 1 (all codes).

**Table 1:** The frequencies of the main codes and subcodes^a^.

Codes			Code description		Depression (n=160), n (%)		Anxiety disorders, (n=393), n (%)		*P* value		χ^2^ (*df*)
**Need for emotional self-expression**											
	All posts	—^b^		13 (8)		22 (6)		.28		1.2 (1)	
	Negative emotions	We categorized posts containing verbal or nonverbal (emoticons) indicators of negative emotions, such as hostility, frustration, and the author’s anger, which were directed toward previous statements, such as “You’re stupid. It’s impossible to read” and “>_<.” However, we did not categorize posts that expressed sadness in the context of empathy or when reflecting the emotions of another forum’s user, such as “I feel sad when I read this.”		8 (5)		10 (3)		.15		2.1 (1)	
	Positive emotions	We categorized posts that contained verbal or nonverbal (emoticons) expressions of joy, gratitude, and pride in response to the content of forum users, such as “(＾v＾)” and “I’m happy when I read that you succeeded.” However, we did not categorize posts that conveyed emotions that hinted at sarcasm or spite, such as “I’m very happy about your misfortune.”		5 (3)		12 (3)		.98		0.0 (1)	
**Need for support (receiving and offering)**											
	All posts	—		116 (72)		246 (63)		.10		2.8 (1)	
	Informational	We categorized posts that discussed symptoms, treatments, and medication side effects based on either book knowledge or personal experience, for example, “Duloxetine can be used to relieve pain,” “I was in treatment for 15 years, no one could help me until I finally tried...,” and “Article 53 of the Mental Health Protection Act states that giving false information about the symptoms of mental disorders is a crime.”		105 (66)		169 (43)		<.001		17.2 (1)	
	Emotional	We categorized posts that showed normalization, compassion, self-esteem support, tension release, hope, motivation, and well wishes, for example, “Don’t give up hope; everyone makes mistakes, but you have many strengths, and you are resilient.”		9 (6)		66 (17)		<.001		11.7 (1)	
	Instrumental	We categorized posts that offered or sought advice on how, when, or where to do something regarding mental health, such as “If you crush the pill first and then wash it down with plenty of water, it will be easier to swallow.”		2 (1)		11 (3)		.27		1.2 (1)	
**Need for social interactions**											
	All posts	—		31 (19)		126 (32)		.004		8.2 (1)	
	Rectification	We categorized posts that involved admitting to a mistake and attempting to resolve a communication misunderstanding with another user, such as “I misunderstood and thought the discussion was about venlafaxine.”		4 (2)		2 (1)		.04		4.1 (1)	
	Disagreement	We categorized posts where users expressed disagreement with others and presented a different opinion, for example, “I disagree; herbal medicine is more effective than drugs.”		2 (1)		7 (2)		.65		0.2 (1)	
	Admitting that someone is right	We categorized posts that demonstrated agreement with another user’s statements and expressed approval, such as “I agree with you” or “B wrote the truth.”		4 (2)		11 (3)		.84		0.0 (1)	
	Community values	We coded posts that promoted attitudes of openness and tolerance and provided information about the community’s values and prohealth behaviors, such as “It’s great to see you back on medication; it will make a difference.” However, we did not categorize posts that advertised products or services, promoted unhealthy behaviors, or expressed sympathy toward someone else, such as “All differences are accepted here.”		3 (2)		13 (3)		.36		0.8 (1)	
	Social life	We categorized posts that encouraged others to share their own experiences, tell jokes, ask questions, and post comments in general, such as “What medications were you taking at that time?” However, we did not categorize posts that focused only on revealing one’s own experiences and exhibited signs of aggression, hostile sarcasm, or irony.		17 (11)		90 (23)		.001		10.4 (1)	
	Private messages	We coded posts that indicated an interaction outside the forum, such as “I sent you a private message.”		0 (0)		1 (0.3)		.52		0.4 (1)	
	Referring to the rules	We coded posts related to forum regulations and what is allowed and not allowed, such as “According to the rules, we cannot post information about drug sales here.”		1 (1)		2 (1)		.87		0.0 (1)	

^a^Differences between forums for people experiencing depression versus anxiety disorders. A *P* value of <.05 was considered significant.

^b^Not applicable.

#### Depression Versus Anxiety Disorders

There was a difference in the dynamics of depression and anxiety forums, as evidenced by the higher number of codes for the identified needs on the anxiety forum than for those on the depression forum (394 vs 160). In general, the most frequently identified needs were the same for depression and anxiety forums and involved informational (105/160, 65.6% and 169/393, 43%, respectively), social life (17/160, 10.6% and 90/393, 22.9%, respectively), and emotional (9/160, 5.6% and 66/393, 16.8%, respectively) needs.

Looking into subcategories of codes, there was a significant difference between depression and anxiety disorders in terms of the need for social interactions (*P*=.004). Forum users expressed their need for informational support and rectification significantly more often on the depression-related forum than on the anxiety-related forum (*P*<.001 and *P*=.04, respectively). By contrast, needs for emotional support and social life were expressed more frequently on the forum for people with anxiety disorders (*P*<.001 and *P*=.001, respectively).

The following needs were expressed more frequently on the forum for depression than on the forum for anxiety disorders: need for expressing anger (*P*=.03), need for information concerning general knowledge or disorders (*P*<.001), need for professional support (*P*=.007), need for information based on own experience (*P*<.001), need for sharing own experience (*P*=.004), and need for the assessment of the situation (*P*=.004).

The posts describing needs for information concerning medications (*P*=.003), hope and motivation (*P*=.02), supporting self-esteem or admiration (*P*=.03), querying or refining (*P*=.005), and joking (*P*=.01) occurred more frequently on the anxiety-related forum than on the depression-related forum.

**Figure 1:**
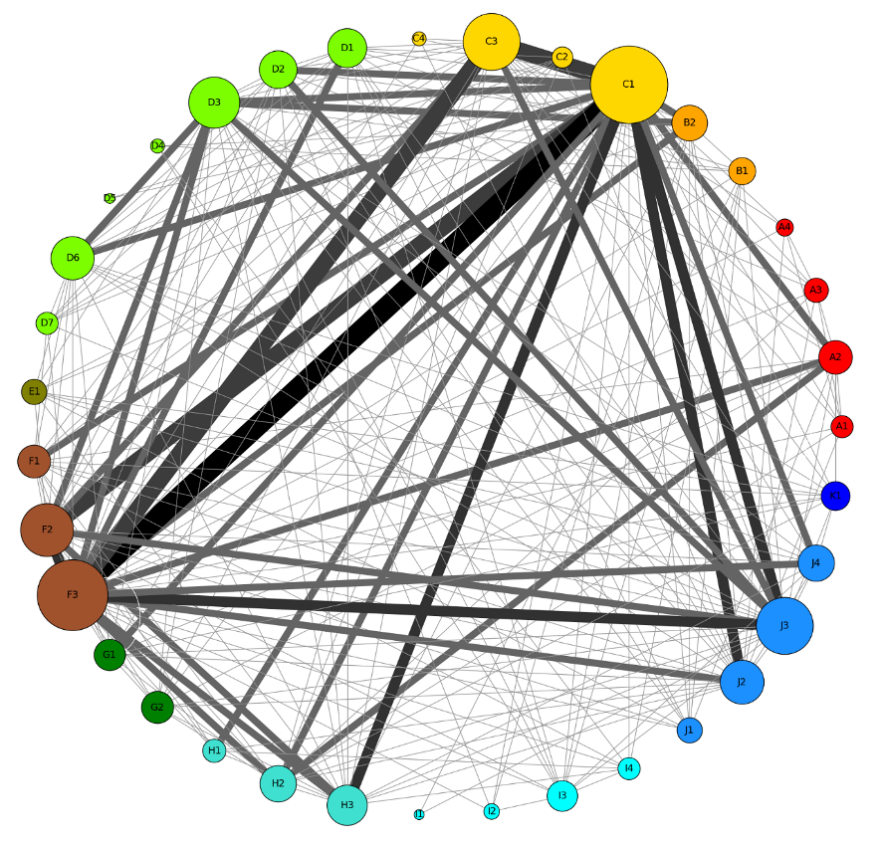
Circular chart of the co-occurrence of needs (component of specific interactions) on both forums together. Node size corresponds to the number of posts where a specific need was expressed. The edges of the graphs indicate co-occurrence with other codes, and their size is proportional to the number of co-occurrences. A1: anger; A2: reluctance; A3: irony and sarcasm; A4: rejection; B1: joy; B2: gratitude; C1: regarding professional support; C2: concerning general knowledge or disorders; C3: concerning medications; C4: regarding adverse effects; D1: I have that too and normalization; D2: wishes; D3: hope and motivation; D4: being with someone; D5: tension release; D6: supporting self-esteem and admiration; D7: compassion; E1: other sources and see more; F1: assessment of the situation; F2: unprofessional information; F3: sharing own experience; G1: instrumental; G2: warnings; H1: rectification; H2: disagreement; H3: admitting that someone is right; I1: acceptance; I2: promotion of expected behaviors; I3: the importance of relationships; I4: mutual protection; J1: encouraging sharing; J2: querying and refining; J3: small talk; J4: joking; K1: referring to the rules.

The co-occurrence of all expressed needs (codes) is shown in Figure 1. The co-occurrence was observed most often for the need for sharing own experience (node F3 in Figure 1) and the need for information regarding professional support (node C1 in Figure 1; 11 times). There was a positive association between these 2 needs (φ=0.966; *P*<.001), which was the strongest positive association noted between 2 interactions.

#### Implicit and Explicit Needs

Differences in the frequencies of expressed needs (explicitly and implicitly) and addressed needs (fully, not fully, directly, and indirectly) between the 2 forums are summarized in Table 2. There were no significant differences between the forums regarding the explicit and implicit ways of expressing the needs (Table 2). The needs were fully addressed more frequently on the depression-related forum than on the anxiety-related forum (Table 2). No other significant differences were observed between the forums in the way needs were addressed (Table 2).

**Table 2:** Frequencies of expressed and addressed needs on the forum for people with depression versus on the forum for people with anxiety disorders^a^.

			Depression forum posts, n (%)		Anxiety disorder forum posts, n (%)		*P* value		*χ*^2^ (*df*)
**Expressing the need^b^**									
	Explicitly	14 (50)		30 (43)		.71		0.1 (1)	
	Implicitly	14 (50)		39 (57)		.58		0.3 (1)	
**Addressing the need**									
	Fully	20 (43)		24 (22)		.01		6.5 (1)	
	Not fully	27 (57)		86 (78)		.08		3.1 (1)	
	Directly	42 (89)		98 (89)		.86		0.0 (1)	
	Indirectly	5 (11)		11 (11)		.88		0.0 (1)	

^a^Differences between forums for people experiencing depression versus anxiety disorders. A *P* value of <.05 was considered significant. Depression: for expressing the need, n=28, and for addressing it, n=47; anxiety: expression, n=69, addressing, n=110.

^b^Every post was coded based on how the need was presented: explicitly (a direct question) or implicitly (a problem disclosed indirectly).

### In-Depth Content Analysis

The analysis suggests that forum users turn to web-based communities to discuss their questions and fears regarding psychiatric hospitalization. The analysis of interactions between forum users revealed 4 mental and emotional representations of a psychiatric hospital: a sense of the unknown, a fear of hospitalization, utterly negative images, and a longing for feeling at home when at the hospital.

#### The Hospital as an Unknown Place

From the users’ posts, an image of a psychiatric hospital as a place shrouded in mystery emerged, with information about it derived mostly from other people, including professionals (Table S2 in Multimedia Appendix 1, quotation 1). This stimulates speculations, fantasies, and various beliefs about the psychiatric hospital. Confusion may generate anxiety among users about what to expect in psychiatric hospitals (quotation 2). Even for people who have been hospitalized many times and have previously collided their ideas about the hospital with reality, hospitalization can be surprising or even scary (quotation 3).

One of the users suggested that he would report his hospitalization on the forum; for him, this was a kind of mission, or a sacrifice, that would provide insights for others into the psychiatric hospital (quotation 4). A decision to go to the hospital was associated with fear, even for one’s own life (quotation 5). The degree of emotional tension before a planned hospitalization is evidenced by the farewell to family, as described by a forum member (quotation 6).

#### The Ambivalence of Presumptions and Needs

Many forum users wrote about the dilemma of whether and when to be hospitalized. The lack of knowledge about what it is like to stay at the psychiatric hospital generated fears fueled by common negative stereotypes. The questions asked by forum members revealed a conflict between these fears and the need to feel safe and cared for. According to the posts, hospitalization was considered necessary when the mental health condition became more severe due to the discontinuation of medication (quotation 7), when the patient posed a threat to themselves or others (quotation 8), when there was a need to verify the diagnosis (quotations 9 to 11), or when the current treatment was ineffective (quotations 12 and 13). The decision of whether to go to the hospital was difficult because users were faced with the need to choose between 2 unappealing options: either take the risk of experiencing the unknown in the hospital or continue to experience emotional problems without any specialist treatment. Doubts were resolved on the forums, where users looked for information about hospitals or asked others to assess whether their condition required hospitalization (quotation 14). A user described hospitalization as a last resort (quotation 15).

#### “Hospitals Are a Total Mess”

Many posts reflected a negative approach to psychiatric hospitals and their functioning or organization. There were 4 areas that stirred particular interest among forum users: accommodation, a community of people (staff and other patients), drugs, and infrastructure. The tone of posts regarding these areas was mostly negative based on the user’s own experience or the experience of family and friends as well as beliefs and emotions. This increased anxiety, shock, and fright and generally made users less inclined to go to the hospital.

Some users described their stay in the hospital, and many responders expressed interest in the admission procedure. Numerous posts indicated that forum users do not know the indications for involuntary hospitalization (quotation 16). Some forum members described scenarios in which the freedom to decide about oneself could be taken away, but others would not accept them. Those who did not agree with suggestions of forced admission maintained that individuals had an unconditional right of self-determination in any situation (quotations 17 and 18). A total of 2 users described situations where they were transported to the ward by the police (quotation 19). They shared traumatic memories and compared the situation to the apprehension of a criminal by the services or a deportation to a concentration camp. They felt as if they were treated unlawfully and in an unprofessional manner:

[The doctor] twisted my words and made up psychiatric epithets on the basis of these distortions.

The reported experiences of involuntary admission revolved around the feeling of objectification, the deprivation of basic rights, slavery, and even dehumanization:

They kept [me]...in confinement like some animal.

Quotation 20

In addition, for some users, the work schedule at the ward resembled rigorous training as in the army, with patients being disciplined by orders (quotation 21).

Some users discussed how hospitalization might affect their mental health. Some forum users had expectations of “miraculous healing” that collided with the actual hospital experience of other users in whom symptoms persisted after hospitalization. Failure to get rid of symptoms during hospitalization inclined users to negatively assess the effectiveness of hospitalization or even consider therapeutic effects to be harmful (quotations 22 and 23).

People associated with the psychiatric hospital, both the staff and other patients, were an important topic among users when sharing observations from the hospital stay or beliefs not supported by their own experience. When writing about hospitalized individuals, users focused mainly on how different patients in closed wards were. These patients were described using vivid imagery and stereotypical representations of a psychiatric hospital popular in the media, which bring to mind a dehumanized image of a patient who is constantly in a straitjacket (quotation 24), is “dependent” (quotation 25), or gets into fights (quotations 26 and 27). Patients may fear that they will be seen as “criminals” who are “constrained for months” or that they will be harmed by “dangerous patients” (quotations 28 and 29). Such beliefs were shared by forum users themselves. They devalued other patients by calling them “freaks” or “plants that are soiling themselves” (quotations 30 and 31).

Medical care in the hospital was considered insufficient. In the opinion of forum users, clinicians do not work hard enough because they focus on more profitable activities in the private sector (quotation 21). According to some forum users, the staff lacks basic empathy and understanding regarding how difficult it is for their patients. One of the forum members suggested that to overcome these gaps, patients and specialists should switch roles (quotation 32).

Forum posts also revealed the anticipation of the stigma associated with psychiatric hospitalization. Users experienced it on many levels, for example, from staff, colleagues, or even themselves. Some users expressed concerns that they will experience prejudice due to common stereotypes around psychiatric hospitalization. One such stereotype is that people with mental disorders require special care and even control because of their unpredictable behavior. Patients actually encountered this kind of treatment in hospitals—they described how their right to privacy was restricted or how they were treated like a child who is under control all the time (quotation 33). Their posts revealed feelings of frustration, resentment, and anger. Forum users also described the experience of being stigmatized by hospital staff or hearing insults in the ward (quotations 34 and 35).

Pharmacological treatment was the third domain where the need for information support was revealed on the forum. Some users wrote about unclear restrictions on their access to medications in a place where, in their opinion, these medications should be provided. They described helplessness and that they had to humiliate themselves and sometimes even beg for medicines that they believed would help them (quotation 36). By contrast, some forum users pointed out that there may be a tendency to solve all the problems of the patient with drugs, without digging into the cause of the condition. When one set of drugs does not help, it is replaced with another and then with another and yet another, which builds an impression that physicians, due to their powerlessness or incompetence, have to experiment on the patient by trial and error (quotations 37 and 38). In addition, there was a feeling of being misunderstood by the staff, for whom the only method to deal with the patient’s difficult emotions was to suppress them (quotation 37).

One of the posts indicated that the patient did not trust the staff. When symptoms became unbearable, the patient could not count on the help of specialists, for example, to be given a medication as needed, but they had to manage on their own and use their hidden medications (quotation 39).

Finally, another aspect of the psychiatric hospital that was negatively represented on the forum was poor infrastructure. The posts revealed that hospitals are short not only of amenities but also of basic equipment for everyday functioning, such as electricity (quotation 40). From what users shared on the forum, hospitalization appeared to be a dehumanizing experience due to “shitty food” and the fact that patients were placed in dark, dingy dormitory rooms “without handles” (quotations 41-43). Moreover, the hospital was viewed as a place that does not provide a sense of security, either regarding one’s health and life (quotation 44) or regarding personal belongings (quotation 45). The extent of negative experiences and the lack of hope that hospital conditions would improve caused anger among forum users. One of them said that only the demolition of the hospital and building it from scratch might improve the current situation (quotation 46).

#### “Almost Like Home”

In contrast to the negative images of a psychiatric hospital that were predominant on the forum, there were also statements of people with positive experiences with institutional care. A user said that he felt “almost at home” in the hospital (quotation 47). Some people voiced their disagreement with negative statements. Users described a hospital that had a busy schedule in terms of classes and therapy and provided round-the-clock specialist care (quotation 48), and patients could feel that their private belongings were safe (quotation 49). Other users pointed out that although patients have to wait for a place in the ward, hospitalization offers various interesting activities (quotation 50) and helps reduce symptoms (quotation 51), establish a diagnosis and adjust medication (quotation 8), or provide a sense of control over medication (quotation 52). Users underlined the unique experience of staying at open wards and described them as pretty and a place of fun where one can feel as in a sanatorium or at a summer camp where discos take place (quotations 53 and 54).

However, another advantage of hospitalization that users mentioned in their posts was the opportunity to make new friends and share experiences with patients who are no different from other people and might be cool (quotation 55). According to some other people, the hospital has an advantage over outpatient treatment, for which there is a long waiting time. In the hospital, medications can be administered as needed and modified very quickly depending on the patient’s current mental state. This can accelerate recovery and is in line with the principles of patient-centered care (quotation 56).

#### Building Bonds

Our analysis showed that the forum enhances the mechanisms that facilitate building a community based on giving hope, motivating others, and showing gratitude and admiration (quotations 4 and 57-61), for example, in response to posts expressing a fear of the first hospitalization in a psychiatric hospital (quotations 57 and 62) or doubts about the treatment received (quotation 63). Some users used irony to tighten bonds on the forum or reduce tension caused by the descriptions of hospital admission (quotation 64) or facing an unacceptable diagnosis during hospitalization (quotation 65). Moreover, some forum users were irritated when others described a division into “better and worse hospitals,” which might be associated with a sense of injustice related to “better and worse treatment” (quotation 66).

## Discussion

### Principal Findings and Context of Other Studies

This study assessed the needs and emotions expressed in web-based forums for people with depression and anxiety disorders, with a focus on the representation of psychiatric hospitals. The most frequently identified needs were the same for depression and anxiety forums and involved informational, social life, and emotional needs. The needs were directly and not fully addressed by forum users. The needs expressed differed between the forums for depression and anxiety disorders, with a more frequent search for emotional support and social life on the anxiety forum and for informational support and rectification on the depression forum. Because anxiety can be accompanied by feelings of restlessness or agitation, individuals who are struggling with anxiety may be more likely to seek out emotional support and social interaction as a way of managing their symptoms [75,76]. They may also be more likely to experience social anxiety or difficulty forming and maintaining relationships, which could further drive their need for emotional support and social connection [77,78]. As for depression, it is often characterized by a persistent negative view of oneself, the world, and the future, which can lead to distorted thinking patterns and difficulty making decisions. Therefore, individuals who are struggling with depression may be more likely to seek out information and guidance that could help them make sense of their experiences and understand their symptoms [79,80]. By contrast, anxiety is often characterized by excessive worry or fear, which can lead to rumination and overthinking. As a result, individuals who are struggling with anxiety may be less likely to seek out information or rectification, as they may already be overwhelmed by the amount of information and may be struggling to make sense of it [81,82]. Another possible explanation is that depression may be more closely associated with feelings of helplessness or hopelessness, which can drive individuals to seek out information or resources that may help them regain a sense of control over their lives [83,84]. Moreover, individuals with depression may be more likely to experience feelings of lethargy or apathy, which could make it more difficult for them to seek out social interaction or emotional support. They may also feel shame or stigma surrounding their condition, which could further discourage them from asking others for help or support [85-87].

We noticed that posts about sharing experiences after staying in the psychiatric hospitals most often occurred together with the need for information regarding professional support. One possible explanation is that individuals who have had similar experiences with mental health issues may be more likely to seek out information and support from mental health professionals. They may also be more willing to share their experiences with other people who are going through similar struggles and may be seeking advice or guidance [88-90]. In addition, it is possible that individuals who have shared their own experiences may have identified gaps in their knowledge or understanding of mental health issues and, therefore, may be more likely to seek out information and support from mental health professionals [91-94].

Psychiatric hospitalization is an important step in the treatment of some patients. Our qualitative analysis revealed that forum users turn to web-based communities to discuss their questions and fears regarding psychiatric hospitals. Moreover, it revealed 4 mental and emotional representations of psychiatric hospitals: a sense of the unknown, a fear of hospitalization, utterly negative images, and a longing for feeling at home.

Many authors of the posts analyzed in this study had a dilemma of whether and when to go to a psychiatric hospital. The dilemma was caused by the lack of knowledge on what hospitalization looked like and what to expect. This increased the level of fear and anxiety.

Our analysis revealed that there were many negative stereotypes about psychiatric hospitals, and most posts about hospitalization were written in a negative tone. This caused greater anxiety, shock, and fear and made users less willing to decide on hospitalization. The perception of hospitalization is influenced by numerous factors, and despite obvious benefits, patients are often dissatisfied with their stay at the hospital [95]. The common reasons for negative experiences include safety concerns, unpleasant communication with hospital staff, and the use of coercive measures [96-98].

Each hospitalized patient should feel safe during their stay at the hospital; however, this is often not the case. It was reported that hospitalization in a closed ward was associated with poor patient satisfaction, which may be linked to restrictions on freedom and the use of coercive measures [99,100]. If coercive measures are used, the patient may feel a sense of powerlessness and humiliation and develop posttraumatic stress disorder [100]. Importantly, this reflects on the relationship with the hospital staff, which is critical for the healing process [99].

The negative perception of hospitalization in a psychiatric hospital may result from stereotypes perpetuated in the media and society that have been consistently harmful and imprecise [101]. This refers, among others, to movies and stories in which psychiatric hospitals are presented as scary and haunted, while people with mental illness are presented as violent and unstable [102]. The way mental illness is described in the media is inaccurate and exaggerated, which only reinforces the stigma [103]. Moreover, a history of psychiatry documented cases of abuse and exploitation, such as locking political prisoners in psychiatric hospitals in the Soviet Union regime [104].

The negative perception of psychiatric hospitalization may also stem from the fact that many psychiatric hospitals are housed in old and unwelcoming buildings, as is the case in many Central European countries, such as Poland, where psychiatric care has been underfinanced for decades [105,106]. Funds for the public sector of psychiatry are extremely low, resulting in understaffing, limited resources, and the lack of modern amenities [107].

The low effectiveness of treatment in psychiatric hospitals can be linked to multiple rehospitalizations [108]. This can be caused by various factors, such as inadequate follow-up care, lack of community resources, and insufficient support for patients after discharge [109]. The issue of psychiatric hospital readmissions is strictly related to the health care system and other contextual factors, such as the financing system and governance structure [110].

One of the important topics that emerged from our analysis was the relationship between patients and staff members. It was reported that maintaining high-quality relationships facilitates the inpatient care pathway and contributes to a reduction in the use of coercive measures [111]. The lack of meaningful communication, absence of regular ward staff, poor staff attitude, and inconsistent and abusive behavior of staff members are some of the potential barriers for patients. Forcible restraint prevents patients from seeking help earlier and is perceived negatively by most patients [111,112]. Different modes of admission to psychiatric units, including involuntary admission, can raise concerns about personal autonomy and boundaries. Patients may feel devalued and uncertain about their rights and decision-making capacity, which can contribute to negative perceptions [99,113].

Our analysis showed that hospitalization in a psychiatric ward is often associated with stigma. This is in line with other studies by Volpe et al [114] and Mutschler et al [115]. People with a history of hospitalization for mental health issues may feel labeled as “crazy” or “unstable” by others. This can lead to feelings of shame, embarrassment, and isolation and may prevent individuals from seeking help in the future [116].

To combat stigma and promote acceptance, it is important to raise awareness about mental health and the experience of people who have been hospitalized in a mental health facility. This can be achieved via education campaigns, support groups, and other initiatives that seek to reduce discrimination and promote understanding. Successful reduction of stigma has the potential to promote a healthy workplace environment, increase the quality of care, and improve treatment outcomes in individuals with stigmatized health conditions [117].

While there were many negative reflections among forum users, we also identified posts describing positive experiences with psychiatric hospitalization. This was linked, among other factors, to the concept of peer support, in which individuals with similar experiences help other patients. This observation was in line with previous research by Katsakou and Priebe [113]. The sense of community can be fostered by psychoeducation programs run by patient-led organizations and psychiatric rehabilitation organizations [118,119]. Peer support can help patients feel less lonely in their struggles and give hope for recovery. In addition, as these programs are often run by individuals who have firsthand experience with hospitalization, they can offer valuable insights to patients and their families. By incorporating peer support and community building into psychiatric care, patients can feel more empowered on their journey to recovery and less stigmatized by their hospitalization experience [120].

### Limitations

This study has several limitations. We identified needs only based on the experiences described by forum users, which may not represent the full spectrum of needs. Other needs may not have been expressed because users either found them difficult to articulate or projected them onto others. There may also be groups of people who have unmet needs but do not have access to web-based forums. The anonymity of the forums made it challenging to verify the identity of users or collect demographic data.

### Future Studies

Nevertheless, our content analysis revealed the diversity of ways in which individuals present their experiences from various events of the treatment trajectory. The findings of this study can serve as a starting point for more in-depth research that considers narrative development (sequence of posts) and collects more detailed data on users’ hospitalization experiences. As our results indicate, there are similarities and unique needs in the 2 groups of the analyzed population. Because there is a lack of studies considering people experiencing psychiatric diagnoses other than depression and anxiety via social media and forums, we believe it would be beneficial to explore the needs of these patients. It may contribute to a more comprehensive understanding of mental health experiences and facilitate more inclusive and effective mental health support systems.

### Conclusions

Web-based forums offer a platform for people experiencing depression and anxiety disorders to express their needs and engage with other individuals who seek information on psychiatric hospitalization. Our study demonstrates that web-based forums can be used by individuals with depression and anxiety disorders to express a wide range of needs. Most needs were addressed by peers but not sufficiently. Our study revealed that forum users share fears and stereotypes regarding hospital stay. Our findings can help mental health professionals understand their patients’ needs and fears, thus guiding the choice of the most appropriate treatment. Using a patient-centered approach, mental health professionals can tailor treatment to address specific concerns and underlying issues, resulting in better patient satisfaction and outcomes. Shared decision-making with patients can ensure the most effective and personalized approach, which can help build trust and establish a therapeutic relationship. This is particularly important for individuals who may be hesitant to seek treatment or have had negative experiences with mental health care. Understanding patients’ needs can help clinicians provide information in a clear and concise language, offer visual aids, refer to reliable sources, and encourage questions. It is also important for clinicians to be cautious of the resources they rely on to avoid misinformation. By adopting these strategies, clinicians can improve the health literacy of patients, thus encouraging them to make informed decisions and take better care of themselves. We believe our findings could hold significant implications for mental health policy makers as well. By gaining insights into the needs of patients, policy makers can shape health care systems and policies to better support patient-centered care. Allocating resources toward addressing barriers, such as financial constraints or logistical difficulties, can enhance access to mental health services, ensuring that individuals receive timely and appropriate treatment. Furthermore, policies aimed at promoting education and awareness about mental health conditions can help reduce stigma and encourage individuals to seek treatment without hesitation.

## Appendix

Multimedia Appendix 1 Frequencies of all codes and quotations from users’ posts on the forums for people with depression and anxiety disorders.

## Data Availability

All data generated or analyzed during this study are included in this published article (and its supplementary information files).
