# Supplementary material for: Needs Expressed in Peer-to-Peer Web-Based Interactions Among People With Depression and Anxiety Disorders Hospitalized in a Mental Health Facility: Mixed Methods Study
Source: J Med Internet Res. 2024 Jul 12;26:e51506. doi: 10.2196/51506 (PMC11285086; doi:10.2196/51506)
Supplement: Multimedia Appendix 1 [file jmir_v26i1e51506_app1.docx]

Supplementary Material

Table S1. The frequencies of all codes.

| Codes | Depression (n, %) | Anxiety disorders (n, %) | p value |
| --- | --- | --- | --- |
|  | n=160 | n=393 |  |
| Need for emotional self-expression | 13 (8) | 22 (6) | 0.283 |
| Negative | 8 (5) | 10 (3) | 0.147 |
| Anger | 2 (1) | 0 | 0.027 |
| Reluctance | 3 (2) | 4 (1) | 0.420 |
| Irony and sarcasm | 2 (1) | 5 (1) | 0.976 |
| Rejection | 1 (1) | 0 | 0.118 |
| Positive | 5 (3) | 12 (3) | 0.976 |
| Joy | 1 (1) | 4 (1) | 0.654 |
| Gratitude | 4 (3) | 8 (2) | 0.744 |
| Need for support | 116 (73) | 246 (62) | 0.095 |
| Informational | 105 (66) | 169 (43) | <0.001 |
| Regarding symptoms and treatment | 50 (31) | 95 (24) | 0.114 |
| Concerning general knowledge / disorders | 6 (4) | 1 (0) | <0.001 |
| Regarding professional support | 42 (26) | 62 (16) | 0.007 |
| Concerning medications | 2 (1) | 31 (8) | 0.003 |
| Regarding adverse effects | 0 | 1 (0) | 0.522 |
| Based on your experience | 51 (32) | 69 (18) | <0.001 |
| Sharing own experience | 33 (21) | 43 (11) | 0.004 |
| Assessment of the situation | 6 (4) | 2 (1) | 0.004 |
| Unprofessional information | 12 (8) | 24 (6) | 0.566 |
| Other sources / see more | 4 (3) | 5 (1) | 0.308 |
| Emotional | 9 (6) | 66 (17) | <0.001 |
| I have that too / normalization | 2 (1) | 10 (3) | 0.340 |
| Wishes | 2 (1) | 13 (3) | 0.175 |
| Hope and motivation | 2 (1) | 24 (6) | 0.015 |
| Being with someone | 0 | 2 (1) | 0.365 |
| Tension release | 1 (1) | 0 (0) | 0.118 |
| Supporting self-esteem / admiration | 1 (1) | 16 (4) | 0.033 |
| Compassion | 1 (1) | 1 (0) | 0.514 |
| Spiritual | 0 | 0 | - |
| Instrumental | 2 (1) | 11 (3) | 0.273 |
| Warnings | 0 | 6 (2) | 0.115 |
| Need for social interactions | 31 (19) | 126 (32) | 0.004 |
| Rectification | 4 (3) | 2 (1) | 0.042 |
| Disagreement | 2 (1) | 7 (2) | 0.649 |
| Admitting that someone is right | 4 (3) | 11 (3) | 0.835 |
| Community values | 3 (2) | 13 (3) | 0.358 |
| Acceptance | 0 | 2 (1) | 0.365 |
| Promotion of expected behaviors | 0 | 2 (1) | 0.365 |
| The importance of relationships | 1 (1) | 8 (2) | 0.233 |
| Mutual protection | 2 (1) | 1 (0) | 0.151 |
| Social life | 17 (11) | 90 (23) | 0.001 |
| Encouraging sharing | 4 (3) | 3 (1) | 0.101 |
| Querying/Refining | 5 (3) | 41 (10) | 0.005 |
| Small talk | 8 (5) | 31 (8) | 0.227 |
| Joking | 0 | 15 (4) | 0.012 |
| Private messages | 0 | 1 (0) | 0.522 |
| Referring to the rules | 1 (1) | 2 (1) | 0.871 |
| * Differences between forums for people with depression vs anxiety disorders; a p value of less than 0.05 was considered significant. | | | |

Table S2. Quotations from users’ posts on the forums for people with depression and anxiety disorders.

| Q# | Quotations |
| --- | --- |
| 1 | *Perhaps my doctor got it wrong because he said that an open [ward] means that patients have their rooms open and do whatever they want in their free time, but they are there 24/7. A closed [ward] is one in which the rooms are closed, i.e., the most severe cases, and the day [ward], as you say, classes and you’re free to go home. Roll as you roll, I'll be there 24/7.*  <anxiety disorder, man, occasional user> |
| 2 | *In general, what does such a hospital stay look like? There is a lot of conflicting information on the Internet. I am terribly afraid of it.*  <depression, woman, super user> |
| 3 | *I have been many times. It's always shock and disbelief. It's not easy there.*  <depression, man, regular user> |
| 4 | *I decided to start this topic, even though there were already similar threads. First of all, I would like to ask for permission to lead this topic. I believe this can help people make the difficult decision to go to hospital. I will try to share my impressions from my stay, day by day, what I felt, what I did, what the treatment is all about. This is my first hospital stay, so I don't know what to expect. Describing this whole situation, I will give myself the feeling that my stay will not be in vain. You will be able to judge for yourself whether such a stay makes sense and is effective.*  <anxiety disorder, man, occasional user> |
| 5 | *I don't know how I'm going to survive the drive to the hospital and the admission to the ward itself*  <anxiety disorder, man, occasional user> |
| 6 | *Today is the last day at home. These are difficult times for me. I have to pack, take a bath, shave, say goodbye to my wife and daughter. I will miss them very much, but I am doing this for them too.*  <anxiety disorder, man, occasional user> |
| 7 | *In the case of discontinuation of drugs, most often sooner or later [the patient] returns to the hospital. In extreme situations, it is necessary to place the patient in a closed ward.*  <anxiety disorder, man, occasional user> |
| 8 | *The psychiatric hospital aims to make sure that your condition does not pose a threat to yourself and others, establish a diagnosis, adjust medications.*  <depression, woman, power user> |
| 9 | *I was considering going to the hospital but what would that do? If I go to the doctor every 2-3 weeks, take the prescribed medications and my condition does not change, how will staying in the hospital make me feel better? Will they give me other drugs?*  <anxiety disorder, woman, occasional user> |
| 10 | *I can't think of a better move than going to the hospital. Each problem must start with a diagnosis in order to know how to deal with it. Be brave, the diagnosis itself will bring relief, because not knowing is even more depressing :-).*  <anxiety disorder, man, occasional user> |
| 11 | *I've been to the hospital twice and each time it was different. Depends on the ward. It all depends on how you hit. But generally, there is nothing to be afraid of. They will set you up on drugs, you can meet a lot of people. Usually once in a few days there are community meetings where everyone can say something about themselves, how they feel, etc. Once I was in a hospital in [anonymized] and there was psychotherapy, occupational therapy, walks with a caretaker, TV. The food is usually terrible, it's good when someone visits you with a roll [emoticon]. You can't have cables or a knife, you charge your phone in the office. You can read books all the time. If you feel bad, take a vacation, don't be afraid of the hospital.*  <depression, woman, occasional user> |
| 12 | *Of the drugs, rexetin has been for 7 years. Recently, a psychiatrist stopped it for me because I was putting on weight and, moreover, it was becoming less and less effective. I switched to efevelon, but after 7-8 weeks, no improvement or even worsening. So I stop it and have been taking asertin for 2 weeks. Not much of an improvement either. That's why I chose the hospital. Well, my psychiatrist made me do it. He says I'll have a better chance of recovery there. I'm tired of this whole situation so I put everything on one card. And the symptoms are as I mentioned, panic attacks, constant fear, I'm afraid all the time, dizziness, loss of balance, anorexia, diarrhea, my hands are shaking, only in the evenings I feel normal.*  <anxiety disorder, man, occasional user> |
| 13 | *Indeed, your condition must be so severe that you have decided to go to the hospital.*  <anxiety disorder, man, occasional user> |
| 14 | *I may have mixed up the categories. Sorry if I did.*  *A quick question. Can self-harm, in itself, "send" me to a mental institution? If so, what does it look like roughly?*  *Thank you in advance for your response.*  <depression, woman, occasional user> |
| 15 | *I think I'll go to the hospital as a last resort.*  <depression, woman, occasional user> |
| 16 | *Recently, after a relatively good period, there was a deterioration. I confided in a psychologist about suicidal thoughts. This lady said that if I wanted to, she could arrange for me to be treated in the hospital, because apparently it is her duty to report such things. I said that I did not agree to this. The lady said that she would let me go because we have known each other for a long time, but I should go to the doctor. I did not go to the doctor because I was afraid that the doctor would refer me to the hospital. In January, unfortunately, I will have to go to him for a prescription. Tell me, do I really have anything to fear?*  <depression, woman, super user> |
| 17 | *It seems to me that if they suspect it as a suicide attempt, they can force you to go to hospital, although one of the users of this forum wrote that you can be thrown out of it just as quickly.*  <depression, unknown, guest> |
| 18 | *It also seems to me that today they don't take it from the distributor, I guess you have to agree to it.*  <depression, unknown, guest> |
| 19 | *the police came to me at night because they got a report that I wanted to kill myself, the ambulance took me to the hospital, and in this hospital I would have to sign a consent for treatment anyway*  <depression, man, super user> |
| 20 | *A phone call to the police was enough. They sat for an hour in the apartment and did not see any aggression. She showed them fake documents and persuaded, persuaded them. I was taken to the emergency room. The monkey doctor from the ambulance wrote me a referral on the side of the ambulance after a one-minute examination. They took me to the psychiatric concentration camp in [anonymized]. The doctor was rude, he twisted my words and made up psychiatric epithets based on those distortions. He threatened me if I didn't sign the release. The guy is called [anonymized] (lowercase intended). They kept me locked up like some kind of an animal for three weeks and then threw insults at me because I wouldn't let the staff push me around.*  <depression, man, occasional user> |
| 21 | *That's what I don't like the most about hospitals. I've always liked to sleep in a long time and this morning's drill is wearing me out. Doctors have to make it by 1 p.m. because then they go to private offices.*  <anxiety disorder, man, occasional user> |
| 22 | *I don't want to disappoint you. Before I went to the hospital for the first time, I also thought that I would be healed miraculously there. I was just in the hospital where they didn't steal, it was nice socially but all my symptoms persisted and staying there didn't give me anything, even the medications were wrong. As a result, I ended up in the hospital every few months, a total of 5 stays in 1.5 years. And only the last stay was different in that the doctor agreed to the drug that I wanted because I read about it on the internet. I was discharged on request and took the medication at home and it helped me so much. But the hospital itself – I don’t how it can help.*  <anxiety disorder, woman, power user> |
| 23 | *I admire your attitude, when I was in my hospital my condition worsened.*  <anxiety disorder, unknown, power user> |
| 24 | *he "only" has anxiety disorder, so it’s hardly likely that they put him right away in a closed ward for more severe cases with straitjackets and no door handles ... it would be stupid, because I suspect that he could be discharged in a worse condition :)*  <anxiety disorder, woman, super user> |
| 25 | *I can describe my "impressions" from the stay in a psychiatric hospital, where I was taken because I scared my woman enough to call the police, I was taken to the hospital for observation with my consent... I was there all week, the first day, I had a terrible experience being in such a shock what had happened, I tried to collect my thoughts somehow, but I was disturbed by the surroundings, I was lying in the corridor, full of dependent people, 90% of people are over 50, the remaining 10% are people with some alcohol or drug abuse, unfortunately also at the age of 30-40, thinking that everything is fine with them, they just drank or smoked too much. Also, I didn't even have normal contact with anyone.*  <depression, man, occasional user> |
| 26 | *my things – so what, I take without asking... because you know... what kind of theft is it anyway? What kind of theft is it to get your stuff back anyway? In my opinion none then other patients fighting, because others were also robbed and I have an attack of crying*  < depression, man, power user>> |
| 27 | *I'm involved in a fight between two female patients, one known for insults, the other for texts, and what do I find out*  <depression, man, power user> |
| 28 | *severe cases are in a closed ward, here no one threatens anyone, rather comfort each other*  <anxiety disorder, man, occasional user> |
| 29 | *at our place it is either closed or open, when I was in a closed one, I was with criminals, they normally spoke prison jargon, people were strapped in for months, well I don't have good memories and the open [ward] (daily) is just fun, even nicely decorated...*  <anxiety disorder, man, power user> |
| 30 | *A: there was also coexistence with human plants that were soling themselves or defecating on the floor (at [anonymized], the floor in the corridor and in the bathroom was really covered with faeces), instead of getting myself together after depression and doing anything, working, what I had left was to walk from the corner to corner and so on, for hours, for days, add to this the rudeness of the lower staff and complete dilettantism, stupidity of the higher [staff], I will never recommend anyone to seek help from a "specialist". I heard from patients that [anonymized] in Warsaw is supposedly better, but I strongly advise against [anonymized], especially the [anonymized] ward.*  *B: I will not cuddle with you and pat you on the head again, you have no privilege here and watch your words, because - as I have already mentioned - you are not the only one who is sick here.*  <A: depression, man, occasional user; B: depression, man, super user> |
| 31 | *I'm afraid that on this forum it will be hard for you to find a common language with anyone if you call sick people "nuts" and people dangerous to the environment.*  <depression, woman, regular user> |
| 32 | *honestly? I will never change my mind, for me the staff of Polish psychiatric hospitals are cattle that should one day switch roles with patients so that they can see!*  *how bad it is for patients in their facilities! I wish that for them! because I hate them and despise them immensely!*  <depression, man, power user> |
| 33 | *The fact that you never have this privacy, even in the bathroom and toilet, you are treated like a handicapped child*  <depression, man, occasional user> |
| 34 | *You are one of a dozen or several dozen lunatics (I have heard the staff using this word many times) in the ward*  <depression, man, super user> |
| 35 | *I agree with this stigmatization and exaggeration by doctors.*  <anxiety disorder, man, occasional user> |
| 36 | *Regarding these drugs, I meant that if I begged, I would get it.*  <anxiety disorder, woman, super user> |
| 37 | *You must not forget that you are a patient of a psychiatric hospital, this type of diseases cannot be cured, they can only be treated, but they last until the death of the patient. They can be suppressed by administering various psychotropic drugs, which is what the managing doctors are doing.*  <anxiety disorder, man, occasional user> |
| 38 | *but anyway [the doctor] can only write an urgent referral, i.e. stat, but I already tried to speed up the deadline once and it didn't help sorry, what follows medications sorry but the doctor has no idea how to treat me now I’m getting pregabaline 60mg and 130mg plus duloxetine 90mg and chlorprothixene 5+5+5mg and neurotop 500x2 and nothing helps sorry but there is no improvement so what the doctor can do, really?*  <depression, man, power user> |
| 39 | *I kept my psycho candies in the garden - "a prison yard" under the bandstand. You always had to manage somehow. I had a few things stashed there if I just couldn’t make it anymore.*  <anxiety disorder, man, power user> |
| 40 | *I have been to hospitals where there were no power outlets in the rooms*  <anxiety disorder, woman, occasional user> |
| 41 | *hospital.. where you throw up with their shitty food and shitty treatment and shitty staff!! more than getting help!!!! that's the truth!!!!!! and it's hard for me.*  <depression, man, power user> |
| 42 | *In my head there were locked doors, dark rooms, dingy beds.*  <anxiety disorder, man, occasional user> |
| 43 | *As someone else mentioned, more like in the hospital - boredom and poor food*  <depression, woman, power user> |
| 44 | *I didn't feel safe there, I didn't feel that the doctors and therapists were involved in the treatment. when you got an attack of strong anxiety, they suggested listening to a relaxation tape*  <depression, man, occasional user> |
| 45 | *It is better not to take valuable items to the hospital. I know from autopsy that in psychiatric wards things "like" to disappear.*  <anxiety disorder, woman, occasional user> |
| 46 | *you can't change the hospital in [anonymized] on [anonymized] street, you would have to demolish it and build a new one in another district of [anonymized] with new staff and those old men who work there turn them adrift because does Polish medicine need someone who doesn’t distinguish PTSD from dissociation? and someone who can't see family problems? and how bad it is for the patient to be in the family??*  <depression, man, power user> |
| 47 | *It's better than I expected. In my head I had locked doors, dark rooms, dingy beds. My roommate is a cool guy with similar problems. We have a nice double room. Normal beds just like at home. A sink inside, a wardrobe, a table. Almost like home ;-). You go outside whenever you want. If you want to go to town, they let you go too. Weekend at home, no problem.*  <anxiety disorder, man, occasional user> |
| 48 | *This hospital has a busy schedule as far as I know. Individual and group classes with a psychotherapist, psychological drawing, relaxation, doctor's care 24 hours a day. I know it's best at home, but lying in bed does nothing and I doubt that I will recover overnight. I want to try, what do I care, in the end I will be in the safest place possible for me.*  <anxiety disorder, man, occasional user> |
| 49 | *A: Entering any psychiatric facility with such things is like asking for theft.*  *B: I guess you haven't been to a psychiatric ward, you always have a phone right by your side.... besides, the rooms are locked with a key that you always have with you, e.g. when out for a smoke.*  <A: anxiety disorder, man, power user; B: anxiety disorder, man, occasional user> |
| 50 | *it will definitely have a positive effect, because firstly it will be quite intense and secondly, there will be various interesting activities, being with people after all, etc.*  <anxiety disorder, woman, super user> |
| 51 | *it was my first hospital stay ever. But I don't regret it, I'm glad that I could be there, it gave me a lot.*  <anxiety disorder, woman, super user > |
| 52 | *It helped me, for example, that I was supervised with medication*  <anxiety disorder, unknown, guest> |
| 53 | *But if you meet cool people, you will organize your time in an interesting way, we played netball, watched movies together, went to the lake, to the city, smaller trips, even disco :) as if at a summer camp*  <anxiety disorder, woman, super user> |
| 54 | *I am in [anonymized] and it is more like a sanatorium.*  <anxiety disorder, man, occasional user> |
| 55 | *You can do it because you want to and that's the most important thing.*  *I keep my fingers crossed, the hospital is not bad, you will meet nice people. Don't be negative. Try to treat it as an adventure. Remember that life is a great playground, only with age we forget to play in it :-)*  *Your posts show how much progress you are making.*  <anxiety disorder, man, occasional user> |
| 56 | *Why will hospitalization help me? Because at home I couldn't get out of it on my own, doctor's visits are here every day, the medication is changed immediately, I don't know the name yet, psychotherapy 3-4 times a week and there are people with similar problems around. Let's hope that all this will have a positive effect :-)*  <anxiety disorder, man, occasional user> |
| 57 | *First of all, I would like to thank you all for your support, because it was thanks to you that I was able to make it in difficult times. I left the hospital on Friday.*  <anxiety disorder, man, occasional user> |
| 58 | *And remember - You are the Winner!*  <anxiety disorder, man, occasional user> |
| 59 | *It's great to read the thoughts of a person who is going through the path that I have already walked :)*  <anxiety disorder, man, occasional user> |
| 60 | *I admire your attitude, in my hospital my condition worsened.*  <anxiety disorder, unknown, power user> |
| 61 | *And supporting each other gives us strength to deal with it on a daily basis.*  <anxiety disorder, man, occasional user > |
| 62 | *I will be happy to read your report from your stay and I keep my fingers crossed that everything goes well*  <anxiety disorder, woman, super user> |
| 63 | *I recently doubted that it is possible to heal, but maybe more such posts and I will reconsider my approach. Maybe there's a chance for everyone.*  <anxiety disorder, man, super user> |
| 64 | *a video report would be more interesting, like Big Brother in a mental institution*  <anxiety disorder, unknown, regular user> |
| 65 | *The bushmen from my village made a very different diagnosis if it comes to my being than the specialist expert from the town-village. And whom to believe*  <depression, unknown, guest> |
| 66 | *“it seems that you are in some 'better' hospital, so I wish you the best”*  <anxiety disorder, unknown, super user> |
| To differentiate between the authors of the posts, we categorized them based on several factors, including the forum they posted on (either for individuals with depression or with anxiety disorders), their self-reported gender (man, woman, or unknown), and their level of activity on the forum. We determined the level of activity based on the number of posts published by the user. Users who published 1-500 posts were considered occasional users; 501-1000 posts, regular users; 1001-5000 posts, super users; and over 5000 posts, power users. Users who were not registered on the forum, were anonymous, and it was difficult to identify their activity on the forum were classified as guests. | |
